# Bioelectrocatalysis of Hemoglobin on Electrodeposited Ag Nanoflowers toward H_2_O_2_ Detection

**DOI:** 10.3390/nano10091628

**Published:** 2020-08-19

**Authors:** Ajay Kumar Yagati, Hien T. Ngoc Le, Sungbo Cho

**Affiliations:** 1 Institute of Analytical Chemistry, Chemo- and Biosensors, Universität Regensburg, 93053 Regensburg, Germany; ajay-kumar.yagati@ur.de; 2 Department of Electronics Engineering, Gachon University, Seongnam-si, Gyeonggi-do 13210, Korea; itnh1809@gachon.ac.kr; 3 Gachon Advanced Institute for Health Science & Technology, Gachon University, Incheon 21999, Korea

**Keywords:** hemoglobin, silver nanoflower, hydrogen peroxide, electrochemical, biosensor

## Abstract

Hydrogen peroxide (H_2_O_2_) is a partially reduced metabolite of oxygen that exerts a diverse array of physiological and pathological activities in living organisms. Therefore, the accurate quantitative determination of H_2_O_2_ is crucial in clinical diagnostics, the food industry, and environmental monitoring. Herein we report the electrosynthesis of silver nanoflowers (AgNFs) on indium tin oxide (ITO) electrodes for direct electron transfer of hemoglobin (Hb) toward the selective quantification of H_2_O_2_. After well-ordered and fully-grown AgNFs were created on an ITO substrate by electrodeposition, their morphological and optical properties were analyzed with scanning electron microscopy and UV–Vis spectroscopy. Hb was immobilized on 3-mercaptopropionic acid-coated AgNFs through carbodiimide cross-linking to form an Hb/AgNF/ITO biosensor. Electrochemical measurement and analysis demonstrated that Hb retained its direct electron transfer and electrocatalytic properties and acted as a H_2_O_2_ sensor with a detection limit of 0.12 µM and a linear detection range of 0.2 to 3.4 mM in phosphate-buffered saline (PBS). The sensitivity, detection limit, and detection range of the Hb/AgNF/ITO biosensor toward detection H_2_O_2_ in human serum was also found to be 0.730 mA mM^−1^ cm^−2^, 90 µM, and 0.2 to 2.6 mM, indicating the clinical application for the H_2_O_2_ detection of the Hb/AgNF/ITO biosensor. Moreover, interference experiments revealed that the Hb/AgNF/ITO sensor displayed excellent selectivity for H_2_O_2_.

## 1. Introduction

Hydrogen peroxide (H_2_O_2_) is a reactive oxygen by-product that acts as a key regulator of various oxidative stress-related processes [1,2]. Moreover, it participates in pathways associated with rheumatoid arthritis [3], atherosclerosis [4,5], asthma [6,7], diabetic vasculopathy [8], and many neurodegenerative diseases [9]. Thus, creating a system for the accurate, sensitive, and selective determination of H_2_O_2_ has been the goal of significant research effort [10]. In the past two decades, there has been a growing interest in the development of H_2_O_2_ electrochemical biosensors based on the enzymatic activity of peptide-nanostructure-modified electrodes [11]. The utilization of nanoparticles (such as Au, Ag, Cu, and Fe) for the creation of nanostructures has received much attention because of their unique morphology, grain size, and physical, electrical, and magnetic properties that make them suitable for application in the fields of drug delivery [12], optoelectronics [13], energy storage elements [14], and microfluidics [15]. Many efforts have been made to synthesize materials characterized by structural, compositional, and morphological uniformity, with a high surface to volume ratio allowing their application in various emerging fields [16]. Among these nanomaterials, Ag nanoparticles have been employed in a variety of applications because of their high extinction coefficient, large interfacial surface, and high thermal and electrical conductivity [17,18]. Moreover, Ag nanostructure-based plasmon biosensors have been shown to exhibit higher intensity wavelength-dependent plasmon bands in comparison with sensors based on Au and its conjugate structures [19]. Furthermore, surface modification chemistry has enabled the coupling of different biomaterials and inorganic materials, allowing their extensive utilization in developing biosensors [20,21].

Hemoglobin (Hb) is a Fe(II)-protoporphyrin IX (heme)-containing protein that contains the “globin fold” domain and reversibly binds molecular oxygen. It has a molar mass of approximately 67,000 g/mol and is composed of two a- and two b-subunits, each containing one molecule of heme [22]. Hb is found in human erythrocytes (red blood cells) at a concentration of approximately 30% (w/v) or 20 mM (in heme). Among heme proteins, Hb is routinely used for the study of electron transfer reactions because of its commercial availability and low cost [23]. Because of its inherent peroxidase activity, Hb has been utilized as the basis for several H_2_O_2_ sensors proposed in recent years. The direct electrochemistry of proteins immobilized on an electrode surface has been studied as a way to sensitively detect H_2_O_2_ without the need for an additional electron transfer mediator. Earlier studies have reported the immobilization of Hb on various electrode surfaces, such as glassy carbon electrodes [24], metal oxides [25], nanoparticles [26], carbon dots [27], and graphene [28], for achieving direct electron transfer toward sensing applications. However, there is a constant quest to achieve more efficient biosensors, i.e., characterized by better sensitivity and shorter response times, by employing minimal fabrication steps.

**Figure 1:**
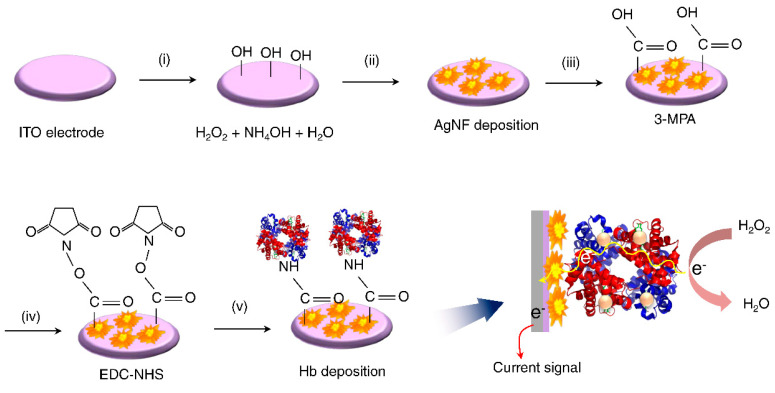
Schematic diagram depicting the surface modifications performed toward the formation of the Hb/AgNF/ITO electrode applied for H_2_O_2_ detection.

In this study, we report the facile electrosynthesis of Ag nanoflowers (AgNF) on an indium tin oxide (ITO) electrode (AgNF/ITO). The surface morphology and the optical characteristics of the AgNFs were examined with scanning electron microscopy and UV–Vis spectroscopy, while the direct electron transfer by Hb immobilized on AgNF electrodes was investigated electrochemically. Based on our results, we propose the development of a sensitive electrochemical H_2_O_2_ sensor based on Hb adsorbed on AgNF-modified ITO electrodes (Hb/AgNF/ITO). Figure 1 shows a schematic diagram summarizing the methodology we employed for creating our proposed modified electrode and its application toward H_2_O_2_ detection.

## 2. Materials and Methods 

### 2.1. Reagents

Human Hb, silver nitrate (AgNO_3_), polyethylene glycol (PEG) 200, *N*-(3-dimethylaminopropyl)-*N*′-ethylcarbodiimide hydrochloride (EDC), N-hydroxysuccinimide (NHS), 3-mercaptopropionic acid (3-MPA), uric acid (UA), L-ascorbic acid (AA), sodium nitrite (NaNO_2_), sodium bicarbonate (NaHCO_3_), and potassium nitrate (KNO_3_) were purchased from Sigma-Aldrich (St. Louis, MO, USA). Triton X-100 was purchased from GeorgiaChem (Smyrna, GA, USA), whereas H_2_O_2_ was obtained from OCI Ltd. (Seoul, Korea) and diluted in deionized water for preparation of the desired molar concentrations. Phosphate-buffered saline (PBS; 10 mM phosphate, pH 7.4) was purchased from BioPrince (Gangwon, Korea). Buffer solutions were prepared using ultrapure deionized water (18.2 MΩ cm^−1^) supplied by a Milli-Q system (Merck, Darmstadt, Germany). All other reagents were of analytical grade or of the highest purity available and used without any further purification unless stated otherwise.

### 2.2. Preparation of the Hb/AgNF/ITO Electrodes

ITO electrodes were initially cleaned by successive ultrasound treatments in Triton X-100: water (1:5, v/v) and ethanol, and were subsequently rinsed with DI water and dried under a N_2_ stream. The substrates were then treated in an oxidizing bath of NH_4_OH:H_2_O_2_:H_2_O (1:1:5, v/v) at 80 °C for 40 min to remove particulate contaminants, washed thoroughly with DI water, and dried in a N_2_ stream.

The electrochemical deposition of AgNFs on bare ITO electrodes was performed in an aqueous solution of AgNO_3_ (1.0 mM in DI water) containing PEG 200 (20 mg/mL) as a surfactant by application of a voltage of −0.9 V against a homemade Ag/Ag^+^ non-aqueous electrode (to avoid the precipitation of AgCl in the solution) for 50 s at a stable temperature of 25 °C. The homemade Ag/Ag^+^ reference electrode was prepared by immersing the Ag wire in a solution of 1 mM of Ag^+^ (an aqueous solution of AgNO_3_) [17]. The deposition time of 50 s was chosen among three tested times (30, 50, and 80 s) because it produced the best results with respect to the formation of fully-grown AgNFs. The presence of PEG 200 acts as a surfactant and mild reducing agent to prevent the aggregation of Ag nanoparticles and to form AgNFs during the electrochemical deposition process. After deposition, the electrodes were cleaned with isopropyl alcohol to remove traces of the surfactant.

The AgNF/ITO electrodes were incubated with 50 mM of 3-MPA for 3 h to allow the formation of a self-assembled monolayer (SAM) from –COOH groups. The 3-MPA-modified electrodes were further incubated with EDC (0.4 M)/NHS (0.1 M) for 40 min (Figure 1). To allow the covalent binding of Hb on the activated surface, 20 µL of a 0.1 mg mL^−1^ Hb solution (pH 7.0) was drop-casted onto each electrode and kept in a humid chamber for 2 h to prevent drying of the surface during binding.

### 2.3. Apparatus and Measurements

UV–Vis absorption measurements on modified electrodes formed on an ITO-coated quartz substrate were performed with an Optizen Pop spectrophotometer (Mecasys, Daejeon, Korea). Both the AgNF substrate and the AgNF electrodes with absorbed Hb were scanned from 300 to 800 nm at a scan speed of 3 nm s^−1^. The EDC–NHS acts as a coupling to activate the COOH group of 3-MPA which binds with AgNFs and provides the strong amide group to link with Hb through the covalent bonding. Since the EDC–NHS coupling can be easily hydrolyzed at room temperature, the UV-Vis of Hb adsorbed AgNF without EDC–NHS coupling was measured. The surface topography of the electrodeposited surfaces was obtained by scanning electron microscopy (SEM) using an EM-30 microscope (COXEM, Daejeon, Korea) operated at a voltage of 20 kV. Surface-enhanced Raman scattering (SERS) is one of most commonly used optical measurement techniques for the in-situ monitoring of organic/inorganic materials at metal–metal and metal/liquid interfaces [29,30]. Ag, Au, Cu, and their conjugations, which are commonly deposited on substrates, produce very strong SERS signals. Thus, this method enables highly sensitive measurements of adsorbed molecules [31,32]. Raman spectroscopy was performed using a UniRAM spectrometer (UniNanoTech, Incheon, Korea) at a spatial resolution of 500 nm in the XY plane and 1 µm in the Z-axis. Spectra were recorded using a laser emitting light at a wavelength of 532 nm. Several scans of 1 s from 500 cm^−1^ to 2500 cm^−1^ were recorded. Averages were calculated and used further. 

Electrochemical measurements were performed with a CHI 660E electrochemical workstation (CH Instruments Inc., Austin, TX, USA) and an IVIUM CompactStat potentiostat (IVIUM Technologies, Eindhoven, the Netherlands) using modified substrate as the working electrode, a platinum wire as the counter electrode, and Ag/AgCl/KCl_sat_ as the reference electrode. Electrochemical impedance spectroscopy (EIS) measurements were performed in 10 mM ferricyanide/ferrocyanide ((Fe(CN)_6_)^3−/4−^) with 0.1 M KCl as the background electrolyte. The input potential for EIS was 10 mV in amplitude with a frequency range of 0.1 to 10^6^ Hz. The electrical properties of the electrodes were modeled with a modified Randles equivalent circuit using impedance-fitting analysis that was performed with the ZView software (Scribner Associates Inc., Southern Pines, NC, USA).

Amperometric (*I*-*t*) measurements on the Hb/AgNF/ITO electrode were performed using various H_2_O_2_ concentrations. The potential was set at −0.5 V and *I*-*t* curves were recorded after successive additions of 10 µL of 100 mM H_2_O_2_ in 5 mL of 10 mM PBS (pH 7.0). Convective transport during amperometric determination was achieved with magnetic stirring at 1200 rpm. The chronoamperogram was recorded with N_2_ purging to circumvent oxygen interference.

## 3. Results

**Figure 2:**
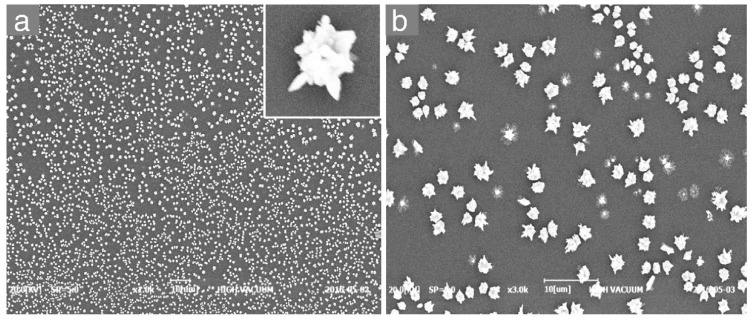
SEM images of (**a**) AgNFs electrodeposited on ITO surface (main) and a single nanoflower (inset), and (**b**) magnified view of the AgNFs shown in (**a**).

Formation of AgNF structures on the ITO electrode by the electrodeposition process was observed by SEM and optical investigation. Figure 2 shows a topographic SEM image of the AgNFs fabricated on the ITO surface, revealing a uniform distribution over the entire surface and a size of around 800 nm. The number density of AgNFs was found to be 147 nanoflowers/µm^2^, which was estimated using ImageJ analysis.

**Figure 3:**
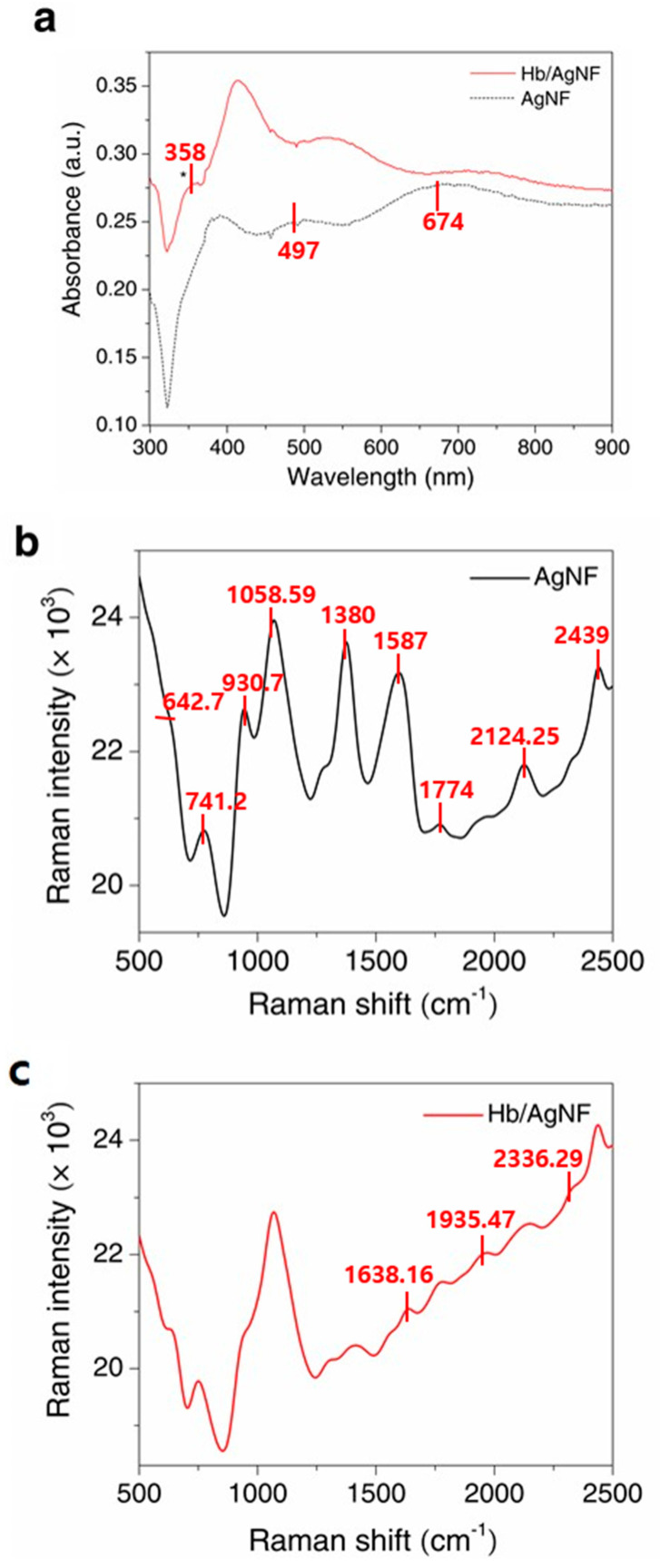
UV–Vis absorption spectra (**a**) and surface-enhanced Raman spectra (**b**) of AgNF/ITO and (**c**) Hb/AgNF/ITO substrates.

Figure 3a demonstrates the UV–Vis spectra of the Hb/AgNF/ITO and AgNF/ITO substrates. The AgNF/ITO substrate exhibits two surface plasmon absorption bands, a dominant broad feature in the visible range (674 nm) produced by the edge of the nanoflower, and a core-produced surface plasmon band at a shorter wavelength (497 nm). The Hb/AgNF/ITO substrate produces an additional shoulder peak at 358 nm caused by the M band of the heme group in hemoglobin, as well as bands corresponding to the electronic transitions of the aromatic amino acids of Hb. Raman spectroscopic analysis was performed on the electrodeposited electrodes, and the resulting spectra are shown in Figure 3b,c. Generally, the intensity of Raman signals is positively dependent on particle size [33], specific surface area [34], and the probability of SERS-active sites [35]. The spectrum of AgNF/ITO electrodes displayed peaks at 642.7, 741.2, 930.7, 1058.59, 1380, 1587, 1774, 2124.25, and 2439 cm^−1^. Moreover, peaks at 1638.16, 1935.47, and 2336.29 cm^−1^ were only observed in the Hb/AgNF/ITO spectrum and seemed to be caused by the vibrational modes of Hb. These data suggest that the silver nanoflower may be a good candidate for the formation of a SERS-active substrate.

**Figure 4:**
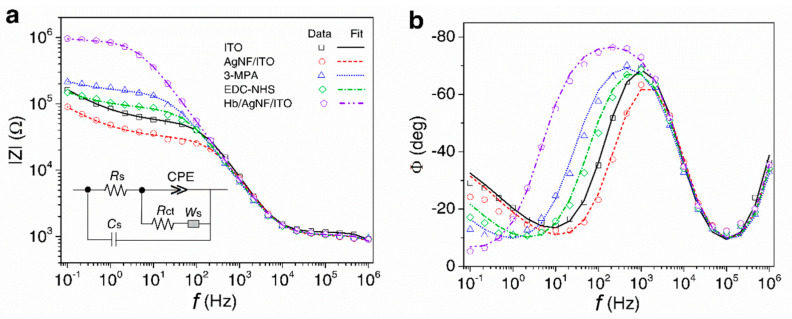
Bode plots of (**a**) impedance magnitude (|Z|) and (**b**) phase (Ф) vs. frequency (*f*) for bare ITO and AgNF/ITO electrodes, 3-MPA self-assembled monolayer (SAM) formation, EDC–NHS activation, and Hb binding. Measurements were performed in 10 mM (Fe(CN)_6_)^3−/4−^ with 0.1 M KCl as background electrolyte. The inset of (**a**) shows the modified Randles equivalent circuit used to fit the measured data.

Electrochemical impedance spectroscopy (EIS) is an efficient analytical method for studying the interfacial properties of a biosensor’s electrode forming elements. Thus, to evaluate the formation of each deposition step toward the Hb/AgNF/ITO electrode, the EIS method was utilized to observe the variation in the impedance modulus of the electrode. Various modified ITO electrodes were analyzed, namely AgNF/ITO, 3-MPA/AgNF/ITO, EDC–NHS/AgNF/ITO, and Hb/AgNF/ITO electrodes. The corresponding Bode plots, in which the frequency was plotted against the impedance or the phase angles of each modified electrode, are presented in Figure 4a,b, respectively. The obtained impedance spectra were fitted to a Randles circuit model shown in the inset of Figure 4a. Stray capacitance (C_S_) was observed at a high-frequency range (above 1 MHz). The ohmic resistance of the electrolyte solution (R_S_), observed in the range of 100 kHz to 10 kHz, did not differ among the tested modified electrodes. The pseudo capacitive characteristics of the electrode interfacial impedance were observed at intermediate frequencies (100 Hz to 10 kHz for all modification processes) and were modeled as a constant phase element (CPE) with an impendence value of 1/(CPE−T·(iω)^CPE−P^) [20], where i is the imaginary unit and ω is the angular frequency. The actual electrode surface modifications could be analyzed at frequencies below 100 Hz, where the charge transfer resistance (R_ct_) corresponding to Warburg diffusion impedance (W_S_) was observed. The value of |*Z*| is dependent on the type of electrode surface modification (Figure 4a), which affects the CPE and *R*_CT_ values of the ITO electrode. The AgNF/ITO electrode had a lower |Z| value, i.e., a higher conductivity, compared to the bare ITO electrode, indicating that the increased surface area of the modified electrode allowed more (Fe(CN)_6_)^3−/4−^ ions to reach its surface. The immobilization of MPA on AgNF resulted in a higher |*Z*| value, indicating the formation of the SAM that prevented (Fe(CN)_6_)^3−/4−^ ions from reaching the electrode. However, the EDC–NHS activation led to a lower |Z| value, because the neutral amine bonds enabled more (Fe(CN)_6_)^3−/4−^ ions to reach the electrode compared to the –COOH groups. Finally, the Hb/AgNF/ITO electrode showed a greatly enhanced |Z|, indicating the complete blocking of (Fe(CN)_6_)^3−/4−^ ions from reaching the electrode. As seen in Figure 4b, the various electrodes also displayed distinct phase (Ф) vs. frequency (f) plots. All types of electrodes (i.e., after any of the used modification processes) can be fitted into Randles equivalent circuits, which are utilized for understanding the interfacial properties of the electrodes [36]. The measured impedance data with fitted results were presented in Nyquist plot of Figure S1. The extrapolated parameters of the Randles circuit for each modification layer are presented in Table 1. The following R_CT_ values were calculated: bare ITO (49.5 kΩ), AgNF/ITO (28.5 kΩ), 3-MPA/AgNF/ITO (149.9 kΩ), EDC–NHS/3MPA/ITO (83.8 kΩ), and Hb/AgNF/ITO (879.7 kΩ).

**Figure 5:**
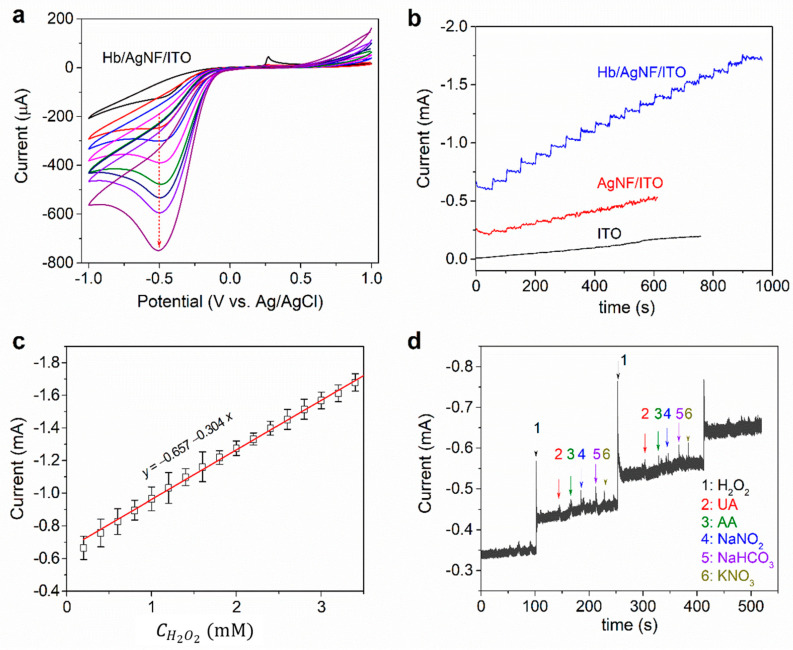
(**a**) Cyclic voltammetry (CV) curves obtained on the Hb/AgNF/ITO electrode upon successive additions of 10 µL aliquots of 100 mM H_2_O_2_ in a 5 mL solution of 10 mM PBS (pH 7.0) at a scan rate of 50 mV/s. The black line of CV presents the blank with no H_2_O_2_. (**b**) Chronoamperometric (CA) curves obtained for bare ITO, AgNF/ITO, and Hb/AgNF/ITO electrodes upon successive additions of 10 µL aliquots of 100 mM H_2_O_2_ to 5 mL of 10 mM PBS with constant stirring (1200 rpm) at an applied potential of −0.5 V vs. Ag/AgCl under nitrogen purging. (**c**) Calibration curve showing the concentration of H_2_O_2_ $\left(C_{H_{2}O_{2}}\right)$ plotted vs. the measured catalytic peak current; data points express mean ± SD of three replicated measurements, and the fitted curve represents a linear fit equation. (**d**) *I*-*t* curves obtained from a Hb/AgNF/ITO electrode at −0.5 V upon successive additions to 5 mL PBS buffer (pH 7.0) of 5 µL aliquots of 0.2 mM uric acid (UA), L-ascorbic acid (AA), sodium nitrite (NaNO_2_), sodium bicarbonate (NaHCO_3_), and potassium nitrate (KNO_3_), along with 5 µL of 0.2 mM H_2_O_2_, with constant stirring.

The electrocatalytic reduction of H_2_O_2_ on Hb/AgNF/ITO electrodes was examined by Cyclic voltammetry (CV), and results are shown in Figure 5a. When H_2_O_2_ was added to an electrochemical cell with a Hb/AgNF/ITO electrode, the reduction peaks were enhanced, whereas the oxidation currents completely disappeared due to an increase in the Hb-catalyzed reduction of H_2_O_2_. The successive addition of 10 µL aliquots of 100 mM H_2_O_2_ to a Hb/AgNF/ITO electrode immersed in 5 mL of PBS (10 mM) resulted in an increment in the cathodic peak current, which was attributed to H_2_O_2_ reduction catalysis taking place on the surface of the modified electrode. These results reveal that the fabricated Hb/AgNF/ITO electrode had an excellent electrocatalytic activity toward H_2_O_2_ detection. The mechanism of the Hb-catalyzed reduction can be broken down into the following reactions [37]:

Hb(Fe^3+^) + H_2_O_2_ → Compound I + H_2_O (1)

Compound **I** + H_2_O_2_→ Hb(Fe^3+^) + O_2_ + H_2_O (2)

Hb(Fe^3+^) + e^−^ + H^+^ → Hb(Fe^2+^) (on the electrode) (3)

Hb(Fe^2+^) + O_2_ → Hb(Fe^2+^)O_2_ (fast) (4)

Hb(Fe^2+^)−O_2_ + 2e^−^ +2 H^+^ → Hb(Fe^2+^) + H_2_O_2_ (on the electrode) (5)

The mechanism includes two catalytic cycles. Hb(Fe^2+^) reacts with O_2_ forming Hb(Fe^2+^)O_2_ (Equation (4)), which can receive two electrons on the electrode surface and revert to Hb(Fe^2+^) (Equation (5)). The H_2_O_2_ produced in Equation (5) can induce or promote the catalytic cycles of Equations (4) and (5). Additionally, CVs of the AgNF/ITO electrode in absence and in presence of H_2_O_2_ are shown in Supplementary Materials Figure S2. CV of AgNF/ITO in the absence of H_2_O_2_ showed the oxidation peak at 0.4 V and broadening reduction peak around −0.4–−0.55 V of silver [38], and the oxidation peak of AgNF/ITO still appeared after 100 mM of H_2_O_2_ was added into PBS solution, indicating no catalyzation of AgNF/ITO for H_2_O_2_.

Chronoamperometric (CA) measurements were performed to construct the current vs. time (*I*-*t*) curves on bare ITO, AgNF, and Hb/AgNF/ITO electrodes in order to elucidate the electrocatalytic response to H_2_O_2_. As seen in Figure 5b, the successive addition of H_2_O_2_ to the Hb/AgNF/ITO electrode results in a clear increment in measured current. In contrast, no significant increment was observed when the same amount was added into AgNF/ITO or bare ITO electrodes. Specifically, bare ITO did not show any catalytic current toward the injected H_2_O_2_, whereas the AgNF/ITO electrode did show some response toward H_2_O_2_. This limited response can be attributed to the fact that AgNFs is an inorganic enzyme mimic displaying peroxidase-like activity [39]. However, the response current soon reached a plateau, and no further activity was observed. We concluded that, among the three electrodes, Hb/AgNF/ITO is the most stable and sensitive sensor of H_2_O_2_ reduction because of the intrinsic activity of Hb toward H_2_O_2_. A response curve (Figure 5c) was obtained for concentrations of H_2_O_2_ between 0.2 mM and 3.4 mM, which fell within the linear range. From the linear regression equation (*y* = −0.304*x* − 0.657; R^2^ = 0.996), the sensitivity (slope of regression curve/area of the electrode) of the Hb/AgNF/ITO electrode toward H_2_O_2_ was found to be 0.956 mA mM^−1^ cm^−2^. The detection limit (LOD) was calculated at 0.12 µM from the equation LOD = *k* × SD_b.g._, where *k* is the signal to noise ratio and SD_b.g._ is the standard deviation of the background signal [40].

To evaluate the practicability of the developed sensor, we checked the electrode’s sensitivity in the presence of various interference compounds, such as ascorbic acid, uric acid, sodium nitrite, sodium bicarbonate, and potassium nitrate, at the concentration of 0.2 mM. The amperometric responses of the biosensor following consecutive injections of 5 µL aliquots of 0.2 mM H_2_O_2_ and aliquots of the above mentioned interfering species are shown in Figure 5d. Measurements clearly show that the interfering species did not influence our biosensor’s sensitivity to H_2_O_2_, indicating its high selectivity for H_2_O_2_. The stability of the sensor was also evaluated by examining its activity for 1 week. Results showed that the sensor retained its activity without degradation in its performance when kept under 4 °C. The density of electroactive species in the surface (Γ_c_) was calculated to 2.2 × 10^−4^ mol/cm^2^ from the slope of peak currents vs. scan rate plot of Supplementary Materials Figure S3 using the peak current equation [41]. Additionally, the Michaelis–Mententen constant (K_m_) of Hb/AgNF/ITO was calculated to 0.63 from the slope of 1/$C_{H_{2}O_{2}}$ (mM^−1^) vs. 1/*I* (mA^−1^) of the Lineweaver–Burk plot [42] in Supplementary Materials Figure S4, indicating the K_m_ of Hb/AgNF/ITO electrode has a lower value as compared to the K_m_ of free enzyme. In the physical meaning, the low K_m_ value represents the high enzyme activity, releasing the high sensitivity of the detection H_2_O_2_, in contrast to the high K_m_ off free enzyme in the comparison [43,44,45,46].

**Figure 6:**
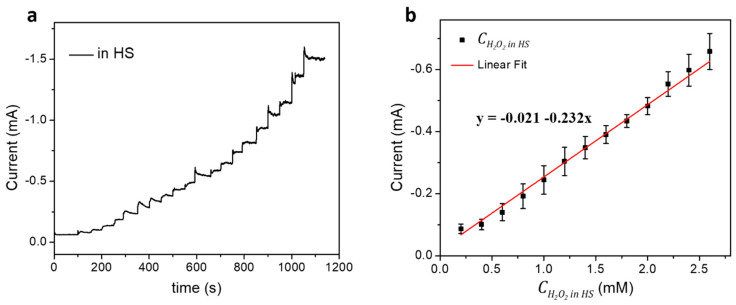
(**a**) CA (*I*-*t*) curves obtained for Hb/AgNF/ITO electrode upon successive additions of 10 µL aliquots of 100 mM H_2_O_2_ in human serum (HS) to 5 mL of 10 mM PBS with constant stirring (1200 rpm) at an applied potential of −0.5 V vs. Ag/AgCl under nitrogen purging. (**b**) Calibration curve showing the concentration of H_2_O_2_ in HS $\left(C_{H_{2}O_{2}\;in\;HS}\right)$ plotted vs. the measured catalytic peak current; data points express mean ± SD of three replicated measurements and the fitted curve represents a linear fit equation.

The clinical application of the Hb/AgNF/ITO sensor toward H_2_O_2_ detection was explored in the human serum (HS) as shown in Figure 6. To avoid the matrix effect, pure HS was diluted 1:200 using 1 × PBS (pH 7.4). Various concentrations of H_2_O_2_ (0.2–4.0 mM) in HS samples were prepared and applied for the CA measurement. Figure 6a shows the increasing of measured current in the addition of H_2_O_2_ in HS to the Hb/AgNF/ITO electrode as well as in PBS (as shown in Figure 5b), indicating the nice electrocatalytic response to H_2_O_2_ in HS of the Hb/AgNF/ITO sensor. A calibration curve was established from a linear range of concentration from 0.2 to 2.6 mM of H_2_O_2_ in HS (Figure 6b) to express a linear regression equation (*y* = –0.021 − 0.232*x*; R^2^ = 0.996). From this, the sensitivity of the Hb/AgNF/ITO sensor toward H_2_O_2_ detection in HS was found to be 0.730 mA mM^−1^ cm^−2^; the LOD was 90 µM, and the detection range was from 0.2 to 2.6 mM. 

Since the reproducibility and repeatability is a crucial factor for clinical application of the sensor, the relative standard deviations (RSDs) of the reproducibility and repeatability of the sensor were evaluated from data measured with three different sensors and from three replicate measurements, respectively. The RSDs of the reproducibility and repeatability of the sensor were found to 4.03% and 3.44%, respectively. The comparison of detection limit, detection range, and sensitivity of the developed biosensor were compared to the diverse nanostructure based-Hb immobilized sensors, as shown in Table 2.

## 4. Conclusions

In conclusion, we developed an easy, simple, and cost-effective method for fabricating Hb/AgNFs/ITO-based H_2_O_2_ sensors with excellent electrochemical catalytic activity and high selectivity. The proposed electrodeposition method achieved the uniform and reproducible formation of AgNFs on the ITO substrate. Optical and electrochemical measurements demonstrated that the AgNF depositions enhanced the measurement sensitivity as compared with bare electrodes. The H_2_O_2_ amperometric sensor exhibited a sensitivity of 0.956 mA mM^−1^ cm^−2^ and a detection limit of 0.12 µM in PBS. The sensor maintained a stable, sensitive, and excellent response for the detection of H_2_O_2_ in the human serum with a sensitivity of 0.730 mA mM^−1^ cm^−2^ and a detection limit of 90 µM. The proposed methodology could prove to be an efficient strategy and a promising platform for the study of protein electron transfer and the development of various biosensors.

## Figures and Tables

**Table 1:** Extrapolated parameters from Randles circuits fitted with impedance data measured at each step of the electrode SAM formation shown in Figure 4a,b.

Electrodes	R_S_ [Ω]	CPE-T (×10^−9^ Ω^−1^ s^CPE−P^)	CPE−P	R_CT_ (Ω)
ITO	1161 ± 10.25	31.97 ± 1.46	0.947 ± 0.005	49,562 ± 594.47
AgNF/ITO	1035 ± 20.74	41.63 ± 4.81	0.921 ± 0.012	28,577 ± 715.05
3-MPA	1049 ± 18.89	78.7 ± 4.96	0.869 ± 0.007	149,970 ± 359.1
EDC–NHS	1040 ± 19.49	64.5 ± 4.82	0.885 ± 0.008	83,884 ± 1932
Hb/AgNF/ITO	1058 ± 12.96	57.9 ± 1.67	0.892 ± 0.003	879,780 ± 15,899

**Table 2:** Comparison of selected quantities measured from Hb immobilized on modified electrodes toward H_2_O_2_ detection.

Modified Electrode Sensors	Applied Potential (V) vs. Ag/AgCl	Detection Limit (µM)	Detection Range (µM)	Sensitivity (µA mM^−1^ cm^−2^)	Reference
Hb/AgNPs BDDE ^a^	−0.4	4.81	500 to 20,000	12.48	[47]
Hb/P123-NGP ^b^	−0.4	8.24	10 to 150	-	[48]
Hb/Au/GR-CS/GCE ^c^	−0.385	0.35	2 to 935	0.35	[49]
Hb/GCFME ^d^	−0.5	2	8 to 214	1400	[50]
Hb-PAN ^e^/GCE ^f^	−0.25	8.3	8.3 to 500	-	[51]
Nafion/Hb/TiO_2_NPs ^g^-rGO ^h^/GCE	−0.35	0.01	0.1 to 140	-	[52]
Nafion/Hb-CS ^i^-bBi_2_S_3_^j^/GCE	−0.4	0.096	0.4 to 4.8	-	[53]
Nafion/Hb/TiO_2_NS ^k^-rGO/GCE	−0.35	0.01	0.1 to 145	-	[54]
Hb/AgNF/ITO	−0.5	0.12 (in PBS); 90 (in HS)	200 to 3400 (in PBS); 200 to 2600 (in HS)	956 (in PBS); 730 (in HS)	This work

^a^ BDDE: Boron doped diamond electrode; ^b^ P123-NPG: Pluronic P123-nanographene platelet; ^c^ GR–CS: Graphene–chitosan; ^d^ GCFME: Graphene modified carbon fiber microelectrode; ^e^ PAN: Polyacrylonitrile; ^f^ GCE: Glassy carbon electrode; ^g^ TiO_2_NPs: Titanium oxide nanoparticles; ^h^ rGO: Reduced graphene oxide; ^i^ CS: Chitosan; ^j^ bBi_2_S_3_: Broccoli-like bismuth sulfide; ^k^ TiO_2_NS: Titanium oxide nanosheets.

## Supplementary Materials

The following are available online at https://www.mdpi.com/2079-4991/10/9/1628/s1, Figure S1: Nyquist plot for bare ITO and AgNF/ITO electrodes, 3-MPA SAM formation, EDC-NHS activation, and Hb binding. Measurements were performed in 10 mM (Fe(CN)_6_)^3−/4−^ with 0.1 M KCl as background electrolyte; Figure S2: CVs of AgNF/ITO electrode in absence and in presence of H_2_O_2_ at a scan rate of 50 mV/s in 5 mL solution of 10 mM PBS (pH 7.0); Figure S3: (a) CV and (b) plot of peak currents vs. scan rate v of Hb/AgNF/ITO in the presence of 10 mM H_2_O_2_ in 10 mM (Fe(CN)_6_)^3−^ with 0.1 M KCl at different scan rate v from 50 to 175 mV/s; Figure S4: Lineweaver-Burk plot of 1/C_H2O2_ (mM^−1^) vs. 1/I (mA^−1^).
