# Supplementary material for: Bioelectrocatalysis of Hemoglobin on Electrodeposited Ag Nanoflowers toward H2O2 Detection
Source: Nanomaterials (Basel). 2020 Aug 19;10(9):1628. doi: 10.3390/nano10091628 (PMC7557759; doi:10.3390/nano10091628)
Supplement: Supplementary file 1 [file nanomaterials-10-01628-s001.pdf]

Bioelectrocatalysis of Hemoglobin on electrodeposited Ag Nanoflowers toward H_2O_2 Detection

Ajay Kumar Yagati [1,†], Hien T. Ngoc Le [2,†] and Sungbo Cho [1,3,*]

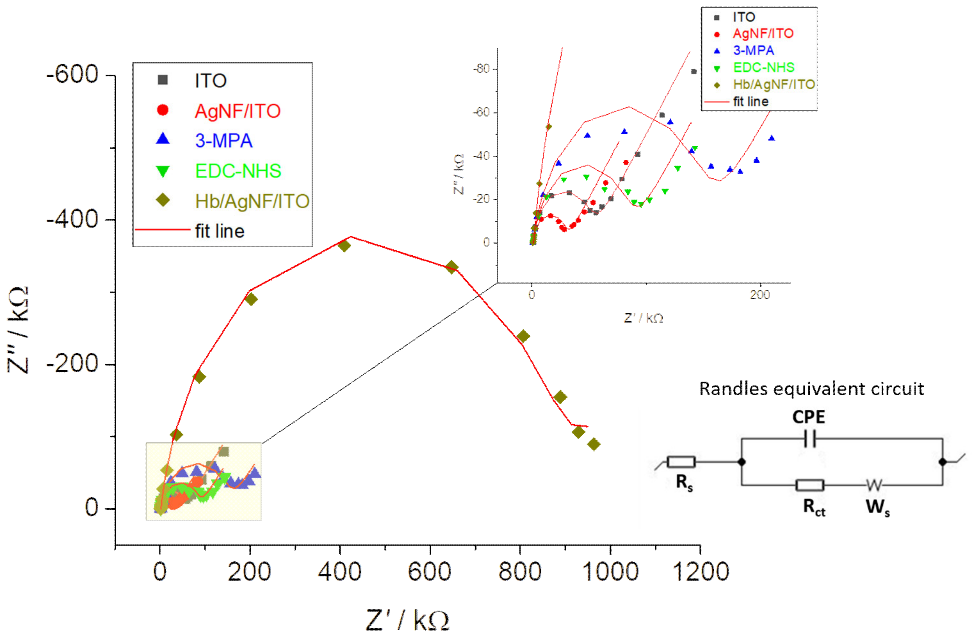

Figure 1. Nyquist plot for bare ITO and AgNF/ITO electrodes, 3-MPA SAM formation, EDC-NHS activation, and Hb binding. Measurements were performed in 10 mM $(Fe(CN)_6)^{3-/4-}$ with 0.1 M KCl as background electrolyte. Inset is Randles equivalent circuit used to fit the experimental data to extrapolate impedance parameters. Here, R_s is the solution resistance, R_{ct} is the charge transfer resistance, CPE is the constant phase element, and W_s is the Warburg diffusion impedance.

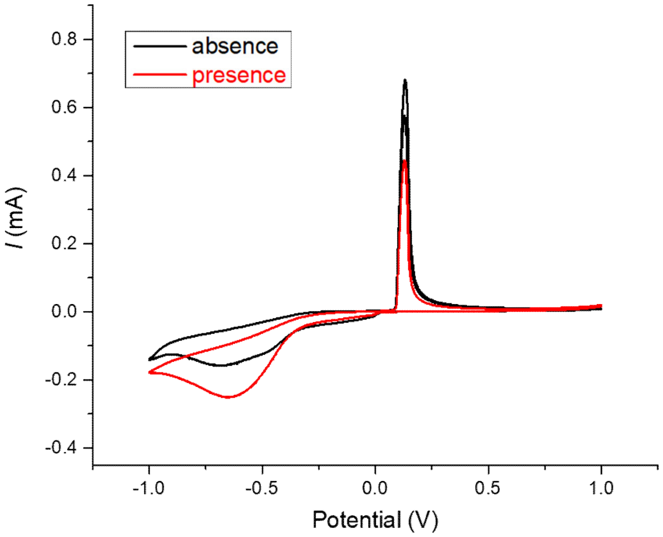

Figure S2. CVs of AgNF/ITO electrode in absence and in presence of H_2O_2 at a scan rate of 50 mV/s in 5 mL solution of 10 mM PBS (pH 7.0).

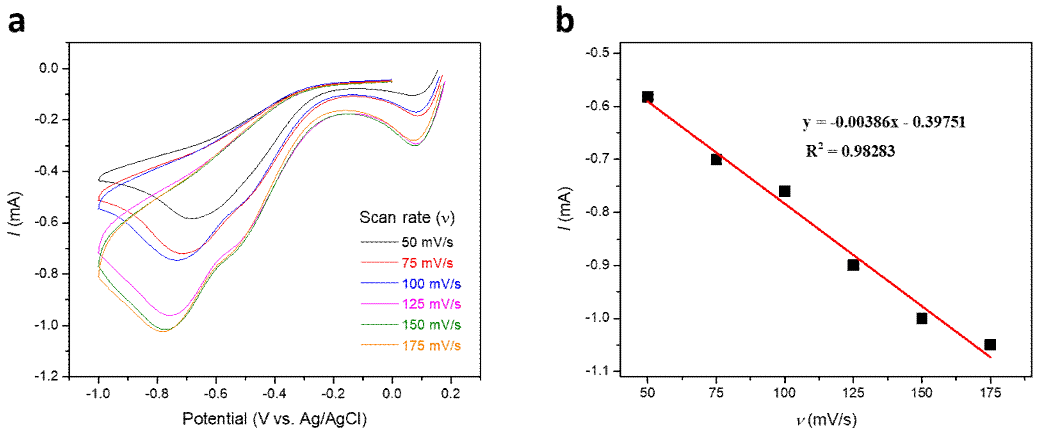

Figure S3. (**a**) CV and (**b**) plot of peak currents vs. scan rate v of Hb/AgNF/ITO in the presence of 10 mM H_2O_2 in 10 mM $(Fe(CN)_6)^{3-}$ with 0.1 M KCl at different scan rate v from 50 to 175 mV/s.

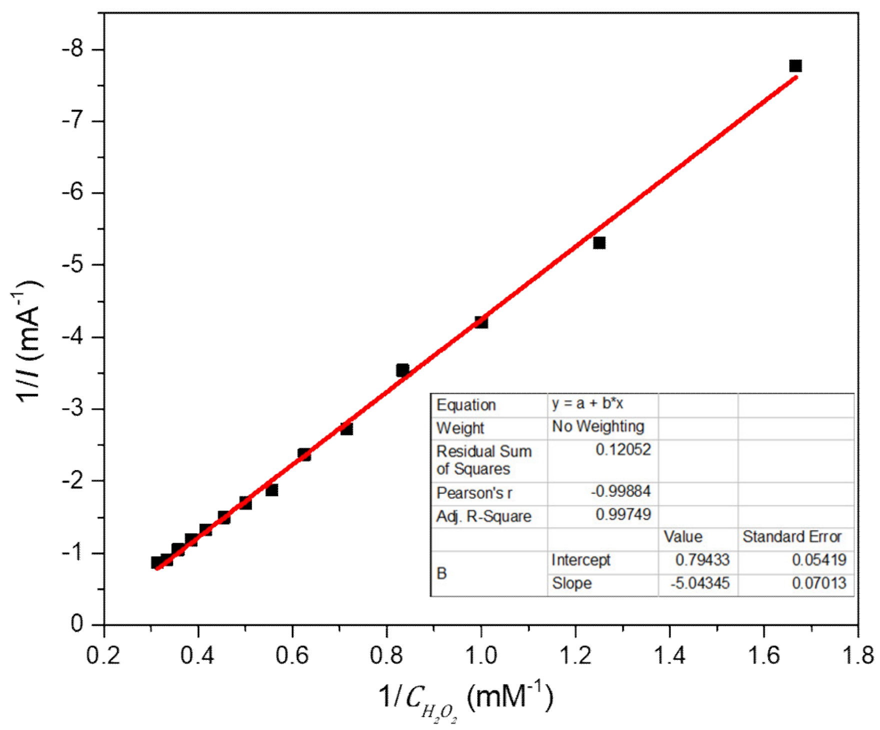

Figure 4. Lineweaver-Burk plot of 1/$C_{H_2O_2}$ (mM^{-1}) vs. 1/*I* (mA^{-1}).
